# Evaluation of Drug–Drug Interactions Between Clarithromycin and Direct Oral Anticoagulants Using Physiologically Based Pharmacokinetic Models

**DOI:** 10.3390/pharmaceutics16111449

**Published:** 2024-11-12

**Authors:** Zhuan Yang, Yuchen Qu, Yewen Sun, Jie Pan, Tong Zhou, Yunli Yu

**Affiliations:** 1 Department of Pharmacy, The Second Affiliated Hospital of Soochow University, Suzhou 215004, China; yangzhuan1018@163.com (Z.Y.); syw0110818@163.com (Y.S.); panzy1122@163.com (J.P.); 2 College of Pharmaceutical Science, Soochow University, Suzhou 215123, China; 3 School of Pharmacy, Nanjing Medical University, Nanjing 211166, China; quyuchen1993@163.com; 4 Department of Respiratory and Critical Care Medicine, The Second Affiliated Hospital of Soochow University, Suzhou 215004, China

**Keywords:** P-gp, DDI, PBPK, clarithromycin, DOACs

## Abstract

**Objective:** This study assessed the pharmacokinetic (PK) interactions between clarithromycin (a P-glycoprotein [P-gp] inhibitor) and four direct oral anticoagulants (DOACs) (P-gp substrates) using physiologically based PK (PBPK) models to elucidate the influence of P-gp in the interaction between them. **Methods:** PBPK models for clarithromycin, DABE–dabigatran (DAB), rivaroxaban, apixaban, and edoxaban were constructed using GastroPlus™ (version 9.9), based on physicochemical data and PK parameters from the literature. The models were optimized and validated in healthy subjects. We evaluated the predictive performance of the established model and further assessed the impact of P-gp on the PK of the four DOACs. Successfully validated models were then used to evaluate potential drug–drug interactions (DDIs) between clarithromycin and the DOACs. **Results:** The established PBPK models accurately described the PK of clarithromycin, DABE–DAB, rivaroxaban, apixaban, and edoxaban. The predicted PK parameters (C_max_, T_max_, AUC_0-t_) were within 0.5–2 times the observed values. A sensitivity analysis of P-gp parameters indicated that an increase in P-gp expression was reduced by in vivo exposure to DOACs. The models demonstrated good predictive ability for DDIs between clarithromycin and the anticoagulants, and the ratio of the predicted values to the observed values of C_max_ and the area under the curve (AUC) in the DDI state was within the range of 0.5–2. **Conclusions:** Comprehensive PBPK models for clarithromycin, DABE–DAB, rivaroxaban, apixaban, and edoxaban were developed, which can effectively predict DDIs mediated by P-gp’s function. These models provide theoretical support for clinical dose adjustments and serve as a foundation for future PBPK model development for DOACs under specific pathological conditions.

## 1. Introduction

Atrial fibrillation (AF) is the most common persistent arrhythmia, with an estimated lifetime risk of approximately 30–40% for White individuals, around 20% for African American individuals, and about 15% for Chinese individuals [1,2]. Currently, direct oral anticoagulants (DOACs) are the standard treatment protocol for preventing stroke in patients with AF [3,4]. Although DOACs have a relatively good safety profile, the increased bleeding risk associated with high drug exposure when a potential drug–drug interaction (DDI) occurs is not negligible [5,6]. For example, in clinical practice, dronedarone was found to double the area under the curve (AUC) and C_max_ of dabigatran (DAB) in vivo, which increased the risk of gastrointestinal bleeding [7].

Patients with AF often take macrolide antibiotics during long-term anticoagulation treatment when respiratory infection occurs [8]. Commonly used macrolide antibiotics, including clarithromycin and erythromycin, are all potent inhibitors of P-glycoprotein (P-gp). Considering that all DOACs are P-gp substrates, predicting potential DDIs between these two classes of drugs is crucial. A study investigating the potential interaction between rivaroxaban and erythromycin found that the combination of rivaroxaban and erythromycin significantly increased the exposure to rivaroxaban in vivo, with the mean AUC_0-inf_ and C_max_ significantly increased by 34% and 38%, respectively [9].

Physiologically based pharmacokinetic (PBPK) models are mathematical models that integrate anatomical and physiological data with drug-related physicochemical properties [10]. They are commonly used for DDI prediction. In this study, we established and validated PBPK models for clarithromycin and the four most frequently used DOACs (i.e., dabigatran etexilate [DABE], rivaroxaban, apixaban, and edoxaban) based on existing in vitro and in vivo data. These validated PBPK models were successfully used to investigate the DDIs between them. Furthermore, we quantitatively analyzed the effects of P-gp’s function on in vivo exposure to DOACs and verified the importance of P-gp in the PK process of DOACs. Our results can provide theoretical support for clinical dose adjustments and lay the groundwork for constructing PBPK models for DOACs under specific pathological conditions in the future.

## 2. Methods

### 2.1. PBPK Model Development

PBPK modeling and simulation were performed using GastroPlus™ (version 9.9; Simulation Plus Inc., Lancaster, CA, USA). The PBPK model development began with an extensive literature search for representative PK studies and compound characteristics, including physicochemical properties, in vitro data, and absorption, distribution, metabolism, and excretion parameters for clarithromycin, DAB, DABE, rivaroxaban, apixaban, and edoxaban. Each PBPK model comprised 14 tissue compartments, including the heart, lungs, brain, fat, muscles, skin, spleen, reproductive organs, gastrointestinal tract, liver, kidneys, yellow bone marrow, red bone marrow, and the rest of the body. Plasma concentration–time data from the literature were digitized using Origin 2021. Table 1 presents the details of all the clinical studies used.

**Table 1:** Clinical trial data used in developing PBPK models.

Substrate	Route of Administration	Dose [mg]	Status	Number	Age Range (Mean) [Years]	Weight Range (Mean) [kg]	BMI Range (Mean) kg/m^2^	Reference
Clarithromycin	iv	250	Fasted	12	21–29 (23.2)	128–176 (153.4)	–	Chu 1992 [11]
	po (tablet, SD)	250	Fasted	17	18–40 (29)	164–188 (175)	–	Chu 1993 [12]
	po (tablet, BID)	250	Fasted	17	18–40 (29)	164–188 (174.9)	–	Chu 1993 [12]
	po (tablet, SD)	500	Fasted	17	20–39 (31)	160–182.9 (174.1)	–	Chu 1993 [12]
	po (tablet, BID)	500	Fasted	18	18–46 (21)	70	–	Sekar 2008 [13]
DAB	iv	1	Fasted	-	26–46 (35)	70–101 (81)	–	Moj 2019 [14]
DABE	po (capsule, SD)	150	Fasted	10	30	70	–	Blech 2008 [15]
	po (capsule, SD)	300	Fasted	10	18–33 (22)	64–82 (75)	–	Delavenne 2013 [16]
Rivaroxaban	iv	1	Fasted	4	21–46 (31.5)	61–94 (78.5)	20.6–30.3 (24.9)	Willmann 2014 [17]
	po (tablet, SD)	5	Fasted	103	19–45(33)	52–106 (81.2)	19.3–31.7 (24.9)	Kubitza 2005 [18]
	po (tablet, SD)	10	Fasted	4	28–54 (43)	60–101 (81.3)	18.1–29.7 (25.4)	Willmann 2014 [17]
	po (tablet, SD)	20	Fasted	22	20–45 (32.9)	62–98 (80.8)	19.4–28.7 (24.4)	Stampfuss 2013 [19]
	po (tablet, SD)	20	Fed	22	20–45 (32.9)	62–98 (80.8)	19.4–28.7 (24.4)	Stampfuss 2013 [19]
Apixaban	iv	2.5	Fasted	9	22–36 (29)	65.7–94.2 (80.6)	20.4–28.1 (25.0)	Charles 2021 [20]
	po (tablet, SD)	10	Fasted	6	21–43 (32)	54–89.2 (74.5)	20.4–29.3 (24.4)	Bashir 2018 [21]
	po (tablet, SD)	20	Fasted	20	21–40 (31)	60–97 (76.8)	20.2–31.9 (26.1)	Charles 2015 [22]
Edoxaban	iv	30	Fasted	-	33.8	80	25.7	Takafumi 2021 [23]
	po (tablet, SD)	60	Fasted	-	33.8	80	25.7	Takafumi 2021 [23]
	po (tablet, SD)	90	Fasted	-	33.8	80	25.7	Takafumi 2021 [23]

### 2.2. Development and Validation of the Clarithromycin PBPK Model

Clarithromycin is a small-molecule compound with good lipid solubility. Thus, the default perfusion-limited model was selected, and the steady-state volume of distribution and tissue–plasma partition coefficients were calculated using the Poulin–Theil homogeneous method. Multiple literature sources indicate that clarithromycin can inhibit CYP3A4- and P-gp-mediated metabolism and transport; however, it is primarily metabolized by CYP3A4, with a small portion excreted through the kidneys [24]. Therefore, P-gp parameters were not set in the transporter module, and the Michaelis constant (k_m)_ and the maximum rate (V_max_) for CYP3A4 were input based on literature reports and IVIVE conversion. Furthermore, a nonspecific renal clearance rate of 7.9 L/h was included in the model. Furthermore, literature reports indicate that clarithromycin has low bioavailability due to a significant intestinal first-pass effect (FPE), with an FPE value set to 25% as determined from the relevant literature [25]. Table S1 presents the specific physiological parameters and clearance rates used in the model. Clinical trial data on the intravenous infusion (250 mg) and oral forms (250 and 500 mg) of clarithromycin were used to evaluate and validate the model’s performance.

### 2.3. Development and Validation of the DABE–DAB PBPK Model

DABE, a small-molecule prodrug, exhibits no pharmacological activity. After oral administration, DABE is rapidly absorbed and hydrolyzed by esterases in the plasma and liver to form DAB. DAB is a potent, competitive, reversible, direct thrombin inhibitor and the main active component in plasma. Therefore, separate PBPK models for DAB and DABE were developed. The models were then integrated by adding DAB as a metabolite of DABE to establish a comprehensive PBPK model for both analytes after the oral administration of DABE. The specific modeling process is outlined as follows.

### 2.4. DAB Intravenous PBPK Model

The PBPK model for DAB following intravenous administration was developed using plasma concentration data to describe the active component’s distribution, metabolism, and excretion. Table S2 lists the specific modeling parameters. Based on Caco-2 measurements, DAB has a low apparent permeability and a high volume of distribution (range = 0.88–1.05 L/kg), suggesting that peripheral tissues are perfusion-limited. The tissue–plasma water partition coefficients were calculated using the Rodgers, Leahy, and Rowland method [26,27,28]. The metabolism module included only UGT2B15-mediated metabolism, with the Michaelis constant (k_m_) and maximum rate values (V_max_) taken from the literature and converted into in vivo UGT2B15 metabolism parameters using the IVIVE module [14]. Drug elimination was set in the kidney compartment at a rate of 7.1 L/h. After inputting the required modeling parameters, plasma concentration–time data for intravenous DAB (250 mg) were placed in the corresponding folder, and a PBPK simulation was performed according to the physiological information of healthy individuals.

### 2.5. Development and Validation of the DABE Oral PBPK Model

The PBPK model for DAB, following the oral administration of DABE, was developed using plasma concentration data to describe its absorption, distribution, metabolism, and excretion. DABE indicated high permeability through Caco-2 cells; therefore, peripheral compartments were also modeled as perfusion-limited tissues, considering the relative volume of distribution of DABE. The tissue–plasma water partition coefficients were calculated using the Poulin–Theil homogeneous method. Unlike DAB, the elimination of DABE primarily occurs through sequential CES1/2 hydrolysis in the liver and intestine [29]. DABE can be hydrolyzed by CES1 to form an intermediate metabolite M1, which is further hydrolyzed by CES2 to DAB, or it can be first hydrolyzed by CES2 to M2 and then by CES1 to the active drug. However, sequential hydrolysis occurs rapidly; therefore, DABE metabolism is considered a single-step hydrolysis process by either CES1 or CES2 [30]. CES2 expression is relatively low in the liver because the enzyme is primarily located in the intestine, whereas CES1 is significantly more abundant in the liver than in the intestine [14]. Therefore, in our PBPK model, CES1 expression is considered in the liver and CES2 expression in the intestine. The CES1/2 metabolites were set as DAB, linking DABE and DAB. Furthermore, DABE has a high affinity for intestinal P-gp, whereas renal P-gp has a minimal effect. Thus, the efflux of DABE by intestinal P-gp must be considered, because it significantly affects DAB exposure in vivo. Table S2 lists the specific modeling parameters for DABE. Clinical trial data on oral DABE (150 and 300 mg) were used to evaluate the model’s performance.

### 2.6. Development and Validation of the Rivaroxaban PBPK Model

Rivaroxaban has a complex dual elimination pathway: two-thirds of the drug is primarily metabolized in the liver via cytochrome P450 enzymes (CYP3A4/2J2), whereas the remaining one-third is mainly excreted unchanged in urine through P-gp [9]. The metabolism and transporter modules in the software express the in vivo and in vitro clearance rates of enzymes and transporters. Because CYP2J2-related data are unavailable by default in the software, they must be manually added to the enzyme expression module, with the healthy adult liver CYP2J2 enzyme content at 1.2 ± 2.1 pmol/mg and the enzyme’s relative molecular weight at 5.77 × 10^−4^ [31,32]. Although no enzymatic reaction kinetics experiments for rivaroxaban have been conducted, the literature suggests an in vitro Km of 10 μmol/L for CYP3A4 and CYP2J2, and the in vitro maximum reaction rate is estimated from the hepatic metabolic fractions of CYP3A4 and CYP2J2, with values summarized in Table S3. Rivaroxaban’s renal clearance involves glomerular filtration and P-gp-mediated tubular secretion [33]. The IVIVE method was used to convert CYP3A4, CYP2J2, and P-gp into hepatic metabolism and renal tubular secretion in healthy adults. Table S3 presents the physiological parameters and clearance rates used in the specific model. Clinical trial data in the literature on the intravenous infusion (1 mg) and oral forms (5, 10, and 20 mg) of rivaroxaban were used to evaluate and validate the model’s performance.

### 2.7. Development and Validation of the Apixaban PBPK Model

Apixaban has a relatively small volume of distribution (0.31 L/kg), good water solubility, and high permeability [34]. The amount of drug distributed to the tissues is primarily limited by tissue blood flow; therefore, the tissue distribution of apixaban was set as a perfusion-limited model. The steady-state volume of distribution and tissue–plasma partition coefficients were calculated using the Poulin–Theil extracellular method [35]. Apixaban metabolism involves CYP3A4/5, CYP2C8, CYP2C9, and CYP2C19, with CYP3A4/5 accounting for approximately 80% of hepatic metabolism [36]. Therefore, the primary enzyme considered was CYP3A4/5, along with the efflux activity of gastrointestinal and renal P-gp. Due to the consideration of renal transporters, the amount of apixaban distributed to the tissues is limited by the transport mechanism and exhibits a certain time dependency. Consequently, the kidneys were set as permeability-limited tissues, and the amount of drug in the kidneys was calculated using the Poulin–Theil homogeneous method. Table S4 presents the specific physiological parameters and clearance rates used in the model. Clinical trial data in the literature on the intravenous infusion (2.5 mg) and oral (10 and 20 mg) forms of apixaban were used to evaluate and validate the model’s performance.

### 2.8. Development and Validation of the Edoxaban PBPK Model

Edoxaban has an oral bioavailability of 62% [37]. It is mainly absorbed in the upper gastrointestinal tract and is primarily eliminated through urinary and biliary excretion, with metabolism contributing minimally to the total clearance. Renal clearance accounts for 50% of total edoxaban clearance [38]. Although P-gp is widely expressed in other tissues, such as the liver and kidneys, the DDI caused by P-gp inhibitors is mainly attributed to inhibition in the gastrointestinal tract [39]. Therefore, in the PBPK model, only intestinal P-gp expression was assumed. Table S5 lists the specific physiological parameters and clearance rates used in the model. Clinical trial data in the literature on the intravenous infusion (30 mg) and oral forms (60 and 90 mg) of edoxaban were used to evaluate and validate the model’s performance.

### 2.9. Evaluation of PBPK Models

The PBPK models were evaluated by comparing the model-predicted plasma concentration–time curves with those observed in actual studies. For PK parameters such as maximum concentration (C_max_) and area under the curve (AUC_0-t_) from dose–time to steady-state tau, the ratio of the predicted values to the observed values from published clinical studies was calculated (defined as fold error [FE]). Predictions were considered reliable and successful when the FE was within 0.5–2.

### 2.10. P-gp Parameter Sensitivity Analysis

Because all four novel oral anticoagulants are substrates of P-gp, their absorption, distribution, metabolism, and excretion may be influenced by P-gp. Our summary of drug physiological parameters highlights the essential role of P-gp. Clarithromycin, a P-gp inhibitor, primarily interacts with DOACs by inhibiting P-gp-mediated absorption, distribution, metabolism, and excretion (ADME) processes. Therefore, a sensitivity analysis of P-gp for the four novel oral anticoagulants was performed to investigate the extent of the effects of P-gp-mediated ADME processes.

### 2.11. Prediction of DDIs

To study potential PK interactions after the coadministration of multiple doses of clarithromycin and single doses of DOACs, clarithromycin’s K_i_ and K_inact_ values for CYP3A4 and P-gp were input into the software’s DDI module, along with clarithromycin’s ka and F. The dosing regimen of clarithromycin and the DOACs, based on actual clinical trials, was then input for dynamic simulation. Population PK simulations were performed based on the characteristics of healthy subjects, with specific details provided in Table 2. The success of the DDI model was evaluated by comparing the model-predicted plasma concentration–time curves with those observed in actual studies, and the FE values of the predicted PK parameters, observed values, and DDI ratios were used to assess the model’s performance.

## 3. Results

### 3.1. Clarithromycin PBPK Model

**Figure 1:**
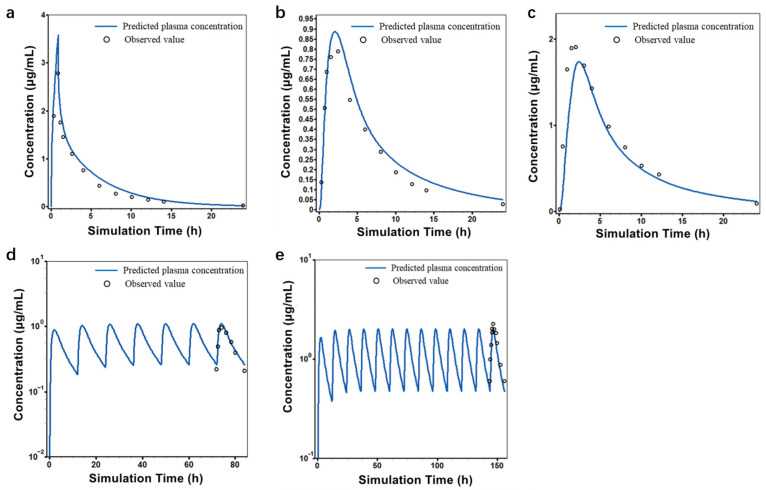
Individually observed (dots) and population-simulated (lines) plasma concentration–time profiles for clarithromycin: a single 30 min IV infusion of clarithromycin at a dose of 250 mg (**a**), a single oral administration of clarithromycin at a dose of 250 mg (**b**), a single oral administration of clarithromycin at a dose of 500 mg (**c**), clarithromycin administered orally multiple times at a dose of 250 mg, BID (**d**), clarithromycin administered orally multiple times at a dose of 500 mg, BID (**e**).

The established PBPK model predicted the PK of clarithromycin in healthy subjects after single (250 or 500 mg) or multiple (250 or 500 mg) oral doses and intravenous administration. As shown in Figure 1, the simulated concentration–time curves closely matched the observed values. Similarly, the predicted C_max_, T_max_, and AUC_0-t_ values matched well with the observed values in all dose studies, with ratios within the acceptable range (Table 3). The successful prediction of multiple oral doses indicated that the clarithromycin model can be used to simulate the scenario of multiple doses in drug combination applications.

### 3.2. DABE and DAB PBPK Models

**Figure 2:**
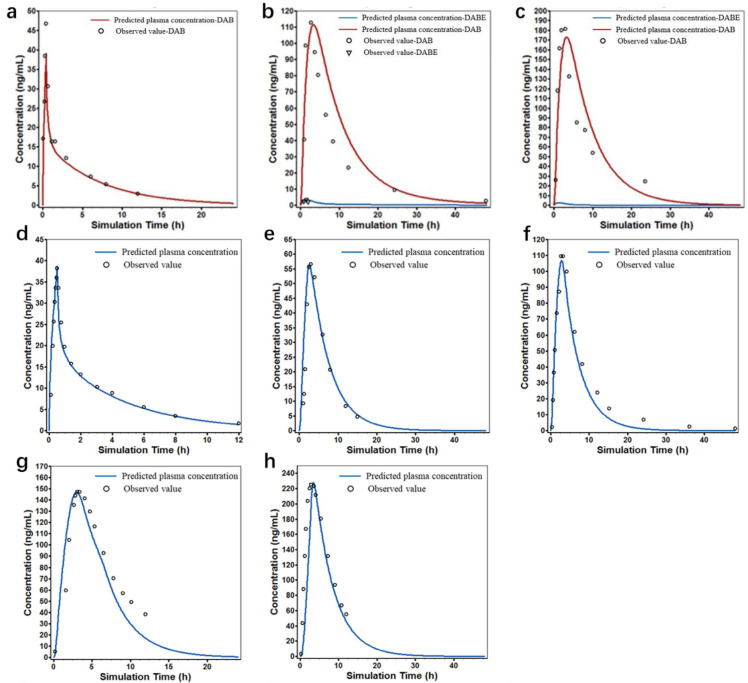

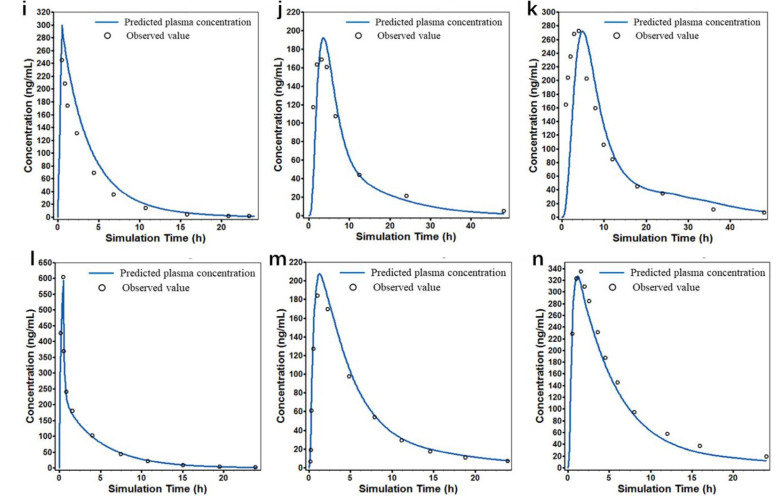
Individually observed (dots) and population-simulated (lines) plasma concentration–time profiles for rivaroxaban: a single 30 min IV infusion of dabigatran at a dose of 1 mg (**a**), a single oral administration of DABE at a dose of 150 mg (**b**), a single oral administration of DABE at a dose of 300 mg (**c**), a single 30 min IV infusion administration of rivaroxaban at a dose of 1 mg (**d**), a single oral administration of rivaroxaban at a dose of 5 mg (**e**), a single oral administration of rivaroxaban at a dose of 10 mg (**f**), a single 20 mg oral dose of rivaroxaban administered in a fasted state (**g**) and a fed state (**h**), a single 30 min IV infusion administration of apixaban at a dose of 2.5 mg (**i**), a single oral administration of apixaban at a dose of 10 mg (**j**), a single oral administration of apixaban at a dose of 20 mg (**k**), a single 30 min IV infusion administration of edoxaban at a dose of 30 mg (**l**), a single oral administration of edoxaban at a dose of 60 mg (**m**), a single oral administration of edoxaban at a dose of 90 mg (**n**).

Table 3 presents the estimated input parameters for DABE and DAB, along with the observed and predicted concentration–time curves shown in Figure 2a–c. The established DAB PBPK model predicted the plasma concentration–time curve of a 1 mg intravenous administration in healthy subjects, as shown in Figure 2a. The FE values for C_max_, T_max_, and AUC_0-t_ were within a reasonable range, indicating that the DAB PBPK model was successfully established. The combined model of DABE and DAB was evaluated by predicting plasma concentration–time curves after the oral administration of 150 and 300 mg DABE in healthy subjects. Figure 2a–c show the model predictions, with the FE values for C_max_, T_max_, and AUC_0-t_ within the classical range of 0.5–2. This indicates the successful establishment of the DABE–DAB PBPK model.

### 3.3. Rivaroxaban PBPK Model

The established rivaroxaban PBPK model predicted the plasma concentration–time curve of a 1 mg intravenous administration in healthy subjects, as shown in Figure 2d–h. The FE values for C_max_, T_max_, and AUC_0-t_ in Table 3 are close to 1, indicating the successful establishment of the distribution and elimination parts of the rivaroxaban PBPK model. The model was further evaluated by predicting plasma concentration–time curves after the oral administration of 5, 10, and 20 mg rivaroxaban in healthy subjects. The FE values for C_max_, T_max_, and AUC_0-t_ also confirmed the successful establishment of the rivaroxaban PBPK model.

### 3.4. Apixaban PBPK Model

The established apixaban PBPK model predicted the plasma concentration–time curve of a 2.5 mg intravenous administration in healthy subjects, as shown in Figure 2i. The FE values for C_max_, T_max_, and AUC_0-t_ also indicated the successful establishment of the distribution and elimination parts of the apixaban PBPK model. The model was further evaluated by predicting plasma concentration–time curves after the oral administration of 10 and 20 mg apixaban in healthy subjects, as shown in Figure 2j,k.

### 3.5. Edoxaban PBPK Model

Table 3 presents the estimated input parameters for edoxaban, along with the observed and predicted concentration–time curves shown in Figure 2l–n. The established edoxaban PBPK model predicted the plasma concentration–time curve of a 30 mg intravenous administration in healthy subjects, as shown in Figure 2l. The model was further evaluated by predicting plasma concentration–time curves after the oral administration of 60 and 90 mg edoxaban in healthy subjects, as shown in Figure 2m,n. The FE values for C_max_, T_max_, and AUC_0-t_ were close to 1, confirming the successful establishment of the edoxaban PBPK model.

### 3.6. P-gp Parameter Sensitivity Analysis

**Figure 3:**
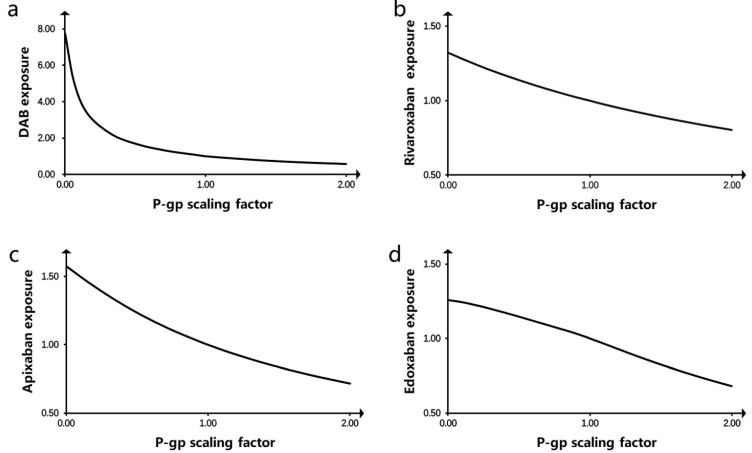
P-gp parameter sensitivity analysis. Presented are P-gp scaling factor vs predicted AUC_0-inf_ for (**a**) DAB, (**b**) rivaroxaban, (**c**) apixaban, and (**d**) edoxaban (baseline = 1).

Figure 3 presents the results of the parameter sensitivity analysis. The horizontal axis represents the magnitude of the P-gp maximum reaction velocity (baseline = 1), whereas the vertical axis represents the change in drug exposure in vivo (baseline = 1). The results indicated that in vivo drug exposure was affected by the P-gp V_max_, which decreased with an increasing V_max_. Therefore, if P-gp is inhibited in vivo, the exposure levels of novel oral anticoagulants will increase.

### 3.7. DDI Prediction

**Figure 4:**
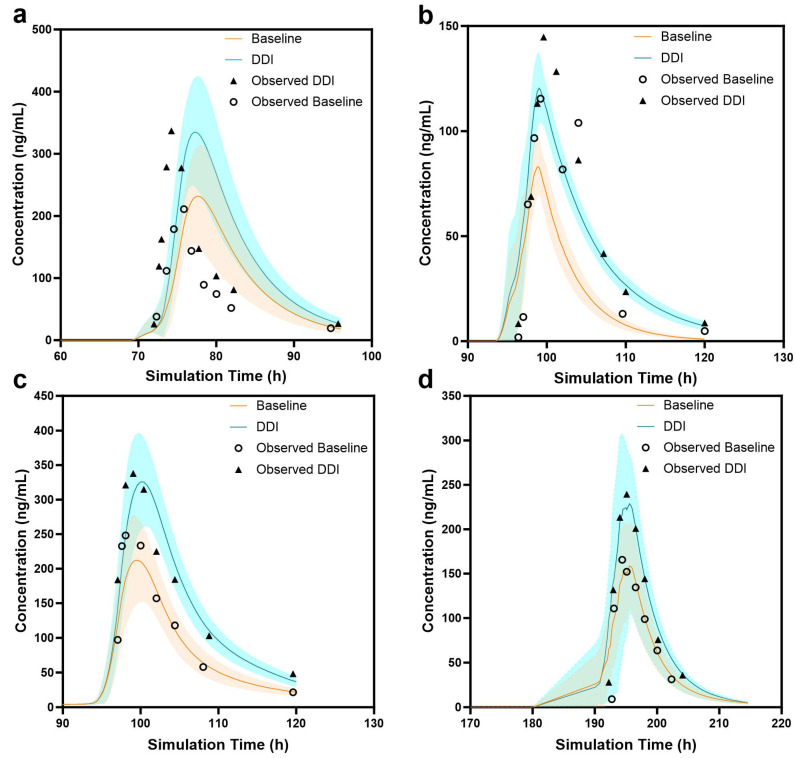
Individually observed (dots) and population-simulated (lines) plasma concentration–time profiles: plasma concentration–time curve of dabigatran (**a**), plasma concentration–time curve of rivaroxaban (**b**), plasma concentration–time curve of apixaban (**c**), plasma concentration–time curve of edoxaban (**d**). Lines represent mean, and error bars represent 90% confidence intervals.

Dynamic simulations of the DDI and independent administration of anticoagulants were performed within the same range of healthy subjects using the previously validated PBPK models. Figure 4 presents the predicted concentration–time curves for the coadministration of clarithromycin with DABE, rivaroxaban, apixaban, and edoxaban. The blue area represents the drug administered alone, whereas the green area represents the combination therapy. Table 4 presents the corresponding PK parameters and FE values.

As shown in Table 4 and Table S6, the coadministration of clarithromycin with the DOACs resulted in C_max_, AUC_0-inf_, and AUC_0-t_ values that were comparable to the results observed in DDI clinical studies.

Clarithromycin with DABE: Compared with the baseline predictions, the DDI prediction showed an increase of 61% in C_max_ and 56.2% in AUC_0-inf_, and the corresponding clinical observation data revealed that the C_max_ increased from 174 ng/mL to 294 ng/mL, indicating that it increased by 68%. The similarity between the predicted and observed results indicated the successful establishment of the PBPK model for the coadministration of clarithromycin and DABE. Meanwhile, we observed that the predicted values were slightly larger than the observed values, which may be due to the random sampling of PK simulation samples in the population, making it impossible to fully reproduce clinical observation values.

Clarithromycin with rivaroxaban: When rivaroxaban was administered alone, the observed C_max_ value was 139 ng/mL, and the observed AUC value was 964 ng·h/mL, whereas, when combined with clarithromycin, the observed C_max_ value was 194 ng/mL, and the observed AUC value was 1469 ng·h/mL, Furthermore, the FE value of the PK parameters was within 0.5–2, which confirmed that the model could effectively predict the PK values of single drugs and drug combination states. Moreover, compared with the baseline predictions, the DDI prediction increased by 42% for the C_max_, which is very close to the results we observed.

Clarithromycin with apixaban: When apixaban was administered alone, the observed C_max_ value was 261 ng/mL, and the observed AUC value was 2531 ng·h/mL, whereas, when combined with clarithromycin, the observed C_max_ value was 339 ng/mL, and the observed AUC value was 4036 ng·h/mL. Both the baseline predicted values and the predicted values under drug combination use were relatively close to the actual observed values. Compared with the baseline predictions, the DDI prediction increased by 74% in C_max_ and 143.6% in AUC_0-inf_. The FE value of both was >1.5. Therefore, the model we established could effectively predict the PK values of single drugs and drug combination states.

Clarithromycin with edoxaban: Whether using the anticoagulants alone or in combination with clarithromycin, the predicted results (C_max_ and AUC_0-inf_) were in good agreement with the observed values and met the DDI acceptance. Compared with the baseline predictions, the DDI prediction increased by 92.9% in C_max_ and 145.9% in AUC_0-inf_. The FE value of both was >1.5. Therefore, the combination of clarithromycin as a P-gp inhibitor and DOACs can increase the in vivo exposure of DOACs, leading to adverse outcomes caused by DDIs.

These results indicated the successful establishment of PBPK models for these combinations.

## 4. Discussion

For patients with AF and acquired pneumonia, macrolides can be administered in combination with DOACs. The DDI caused by multidrug combination therapy increases the exposure to DOACs in vivo and is associated with a high risk of bleeding. This study established and validated PBPK models for clarithromycin, DABE, rivaroxaban, apixaban, and edoxaban based on existing in vitro and in vivo data. The validated PBPK models were used to investigate the DDIs between clarithromycin and the DOACs, with the aim of providing theoretical support for clinical dose adjustment.

In this study, we successfully evaluate the DDIs between clarithromycin and four DOACs by PBPK modeling. The prediction results revealed that compared with the DOACs used alone, combination therapy with clarithromycin increased the AUC_0-inf_ of DAB in vivo by 56%, rivaroxaban by 128%, apixaban by 143%, and edoxaban by 145% and increased the C_max_ of DAB by 61%, rivaroxaban by 42%, apixaban by 74%, and edoxaban by 92%. Furthermore, clarithromycin undoubtedly significantly increased DOAC exposure. As a moderate P-gp inhibitor, clarithromycin significantly affected the metabolic kinetics of P-gp substrates. DOACs are typical P-gp substrates, and P-gp, as the main efflux transporter, is highly expressed in the intestine. Therefore, we conducted a parameter sensitivity analysis on the effect of DOACs on P-gp in the intestine. The results revealed that the in vivo exposure to the four anticoagulants was inversely proportional to the expression level of P-gp. DOAC exposure in vivo increased with a decrease in P-gp expression level. In particular, when the expression level of P-gp decreased by 10% from the baseline value, our results revealed that the AUC of DAB increased to 112% of the baseline value, the AUC of rivaroxaban increased to 103% of the baseline value, and the AUCs of apixaban and edoxaban increased to 103% and 106% of the baseline value, respectively.

The results indirectly proved that P-gp significantly contributes to ADME processes for DOACs. Furthermore, the findings also suggested that the DDIs between P-gp inhibitors and DOACs should be considered. For example, dronedarone is a strong P-gp inhibitor. A retrospective cohort study confirmed that dronedarone doubles the AUC and C_max_ of DAB; thus, its coadministration is contraindicated. A single oral dose of 600 mg amiodarone, a P-gp inhibitor, was shown to increase dabigatran bioavailability by approximately 50–60% in healthy volunteers [42]. Furthermore, a significantly higher incidence of major bleeding was reported after the coadministration of amiodarone in DAB-treated patients than in patients treated with dabigatran alone [43]. Moreover, caution must be observed when administering quinidine and verapamil, which are moderate and mild P-gp inhibitors, respectively.

Many studies have established PBPK models for DOACs; however, the research directions and focus are different. Jennifer et al. evaluated the ability of a PBPK model to predict differences in the magnitude of P-gp DDI between a microdose and a therapeutic dose of DABE to address a specific question of predicting intestinal P-gp-mediated DDI. Takafumi et al. quantitatively evaluated the effect of P-gp efflux on edoxaban absorption in the gastrointestinal tract. Based on these findings, we supplemented and improved the PK effects of P-gp on other DOACs and combined P-gp inhibitors with DOACs to predict and evaluate the effect of clarithromycin on DOAC exposure in vivo. In this study, we focused on intestinal P-gp and horizontally compared the changes in and effects of P-gp’s function on exposure to four anticoagulant drugs. This improves our understanding of the significant contribution of P-gp in DOACs’ ADME in gastrointestinal tracts.

However, our current research still has some shortcomings, because the clinical trial data we used were all from healthy subjects, which resulted in our results being applicable only to healthy individuals. However, the actual clinical application of DOACs will face many complex situations. The exposure of patients with diabetes mellitus, elderly individuals, and individuals with liver and kidney damage after taking drugs to DOACs is often higher than that of healthy individuals. For example, Daniel et al. reported that as renal function decreases, exposure to DAB (AUC and C_max_) significantly increases. Multiple drugs and complications are associated with a higher mortality and bleeding risk in patients taking DOACs. Therefore, for patients with atrial fibrillation, acquired pneumonia, recent surgery, and liver and kidney injury who are taking clarithromycin and DOACs, clinical doctors should be more cautious in selecting medications and monitor the patient condition in real time to avoid bleeding due to DDIs.

Moreover, the PBPK models established in this study for predicting the PK characteristics of DOACs in healthy subjects during coadministration with clarithromycin provide a theoretical basis for the dose adjustment of DOACs. This approach can significantly reduce the incidence of adverse DDIs, thereby enhancing patient safety. The models allow an extension to other special populations (e.g., individuals with renal impairment) by implementing more complex structures for specific organs or relevant processes, which can provide a basis for DDI simulation and prediction in special patients, with important clinical implications.

## 5. Conclusions

In summary, we established PBPK models for clarithromycin and four DOACs. Based on the findings of this study, we accurately predicted the PK profiles of DOACs in vivo when clarithromycin was combined with these drugs. The results revealed that the use of clarithromycin increased the exposure to DOACs in vivo. This is because clarithromycin is a moderate P-gp inhibitor that inhibits the transport of DOACs and their metabolites to the intestine. Its accumulation in the blood increases exposure (AUC and C_max_). When the plasma concentration exceeds the treatment window of the drug, it may bring a certain risk of bleeding to the patients. At this time, the medication dosage should be adjusted in a timely manner according to the patients’ physiological conditions and medication situations to avoid the medication risks caused by DDIs.

## Figures and Tables

**Table 2:** Clinical trial data used in DDI prediction.

Substrate	Inhibitor	Number	Age Range (Mean) [Years]	Weight Range (Mean) [kg]	Height Range (Mean) [cm]	BMI Range (Mean) kg/m^2^	Reference
DABE 300 mg (capsule)	Clarithromycin (tablet)	10	18–33 (22)	64–82 (75)	175–188 (180)	-	Delavenne 2013 [16]
Rivaroxaban 10 mg (tablet)	Clarithromycin (tablet)	16	24–50 (37.6)	81.1 ± 12	-	18–32	Mueck 2013 [9]
Apixaban 10 mg (tablet)	Clarithromycin (tablet)	19	20–44 (31.3)	54.5–96.9 (72.35)	154.3–185.9 (67.65)	20.8–29.3 (25.64)	Garonzik 2019 [40]
Edoxaban 60 mg (tablet)	Clarithromycin (tablet)	12	20–54 (26)	-	-	22.5	Lenard 2024 [41]

DABE: Clarithromycin 500 mg tablet administered orally twice daily (p.o. bid) for 3 days, followed by concurrent administration on day 4. Rivaroxaban/clarithromycin 500 mg tablet given orally twice daily for 4 days, with concurrent administration starting on day 5. Apixaban/clarithromycin 500 mg tablet taken orally twice daily for 4 days, then administered concurrently on day 5. Edoxaban/clarithromycin 500 mg tablet taken orally twice daily for 7 days, followed by concurrent administration beginning on day 9.

**Table 3:** Comparisons between PBPK model predictions and clinically observed data collected from the literature.

Drug	Dose (mg)	C_max_/(ng/mL)			T_max_/h			AUC_0-t_/(ng·h/mL)		
		Observed Data	Predicted Data	FE	Observed Data	Predicted Data	FE	Observed Data	Predicted Data	FE
Clarithromycin	250	2780	3580	1.28	0.86	0.86	1.00	9500	10870	1.14
Clarithromycin	250	790	890	1.13	2.51	2.08	0.83	5710	6730	1.18
Clarithromycin	250	950	1100	1.12	74.27	73.98	1.00	-	48550	-
Clarithromycin	500	1910	1740	0.91	2.06	2.4	1.16	15570	13430	0.86
Clarithromycin	500	2250	2020	0.90	146.8	146.5	1.00	-	172	-
DAB	1	46.67	39.08	0.84	0.44	0.44	1	117.9	121.6	1.03
DABE	150	112.68	111.8	0.99	2.69	3.45	1.28	-	1314.2	-
DABE	300	181.48	173.24	0.95	3.01	3.2	1.06	-	1836.3	-
Rivaroxaban	1	38.15	36.36	0.95	0.5	0.52	1.04	87.00	86.17	0.99
Rivaroxaban	5	56.50	56.37	1.00	3.02	2.57	0.85	374.50	395.2	1.06
Rivaroxaban	10	109.4	106.9	0.98	2.66	2.82	1.06	856.10	736.4	0.86
Rivaroxaban	20 (fasted)	147.3	147.0	1.00	3.07	2.96	0.96	969.40	957.0	0.99
Rivaroxaban	20 (fed)	225.0	228.1	1.01	2.88	3.36	1.17	1618.6	1589.9	0.98
Apixaban	2.5	245.1	299.8	1.22	0.5	0.5	1.00	878.7	1152.7	1.31
Apixaban	10	168.8	192.1	1.14	3.20	3.62	1.13	2014.8	1775.1	0.88
Apixaban	20	272.0	271.8	1.00	4.01	4.9	1.22	3090.2	3136.4	1.01
Edoxaban	30	603.7	593.9	0.98	0.50	0.50	1.00	1259.8	1230.3	0.98
Edoxaban	60	183.9	207.8	1.13	1.06	1.28	1.20	1220	1315.7	1.08
Edoxaban	90	334.1	326.8	0.98	1.58	1.25	0.80	2347.2	2055.3	0.88

**Table 4:** Comparative analysis of DDI predictions for DABE–DAB, rivaroxaban, apixaban, and edoxaban coadministered with clarithromycin.

Predicted Value		DAB		Rivaroxaban		Apixaban		Edoxaban	
		Mean	90%CI	Mean	90%CI	Mean	90%CI	Mean	90%CI
C_max_ (ng/mL)	DDI	359	267–453	152	142–160	355	297–413	293	205–380
	Baseline	249	165–302	108	99–120	240	186–293	198	111–285
	Ratio	1.610	1.345–1.885	1.420	1.275–1.551	1.740	1.470–2.009	1.929	1.427–2.430
AUC_0-inf_ (ng·h/mL)	DDI	3614.6	2750.0–4479.1	1233.7	1095.8–1371.5	3972.0	3478.3–4465.6	2022.8	1625.1–2420.5
	Baseline	2532.9	1757.9–3307.9	594.2	502.6–685.9	2129.3	1649.7–2608.9	1047.7	737.0–1358.4
	Ratio	1.562	1.310–1.813	2.283	1.877–2.689	2.436	1.842–3.029	2.459	1.854–3.064
AUC_0-t_ (ng·h/mL)	DDI	3421.7	2606.4–4237.1	1169.4	1056.5–1282.3	3547.8	3072.2–4023.3	1980.3	1593.7–2366.9
	Baseline	2401.6	1667.0–3136.3	589.8	500.1–679.6	1895.3	1493.8–2296.8	1007.0	694.5–1319.4
	Ratio	1.563	1.311–1.315	2.182	1.803–2.560	2.338	1.829–2.846	2.550	1.923–3.176

## Data Availability

The original contributions presented in the study are included in the article/Supplementary Material; further inquiries can be directed to the corresponding author/s.

## Supplementary Materials

The following supporting information can be downloaded at: https://www.mdpi.com/article/10.3390/pharmaceutics16111449/s1, Table S1: Physicochemical and absorption, distribution, metabolism, and excretion (ADME) information on Clarithromycin; Table S2: Physicochemical and absorption, distribution, metabolism, and excretion (ADME) information on dabigatran etexilate and dabigatran; Table S3: Physicochemical and absorption, distribution, metabolism, and excretion (ADME) information on Rivaroxaban; Table S4: Physicochemical and absorption, distribution, metabolism, and excretion (ADME) information on Apixaban; Table S5: Physicochemical and absorption, distribution, metabolism, and excretion (ADME) information on Edoxaban; Table S6: Comparisons between PBPK model predictions and reported clinical data collated from the literature.
