# Supplementary material for: Evaluation of Drug–Drug Interactions Between Clarithromycin and Direct Oral Anticoagulants Using Physiologically Based Pharmacokinetic Models
Source: Pharmaceutics. 2024 Nov 12;16(11):1449. doi: 10.3390/pharmaceutics16111449 (PMC11597346; doi:10.3390/pharmaceutics16111449)
Supplement: Supplementary file 1 [file pharmaceutics-16-01449-s001.zip › pharmaceutics-3270861-supplementary.pdf]

Evaluation of Drug–Drug Interactions Between Clarithromycin and Direct Oral Anticoagulants Using Physiologically Based Pharmacokinetic Models: Supplementary Materials

Zhuan Yang, Yuchen Qu, Yewen Sun, Jie Pan, Tong Zhou and Yunli Yu

Table S1. Physicochemical and absorption, distribution, metabolism, and excretion (ADME) information on Clarithromycin.

Parameters	Unit	Value	Reference
Structure		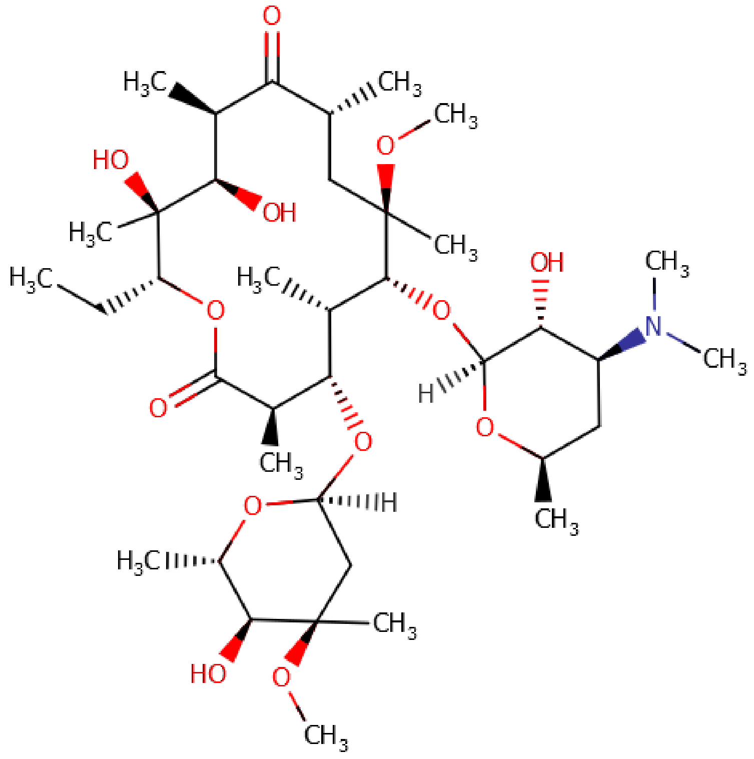	
Mw	g/mol	747.95	Drugbank
LogP	-	2.3	Lappin 2011[1]
Solubility	mg/mL	0.74(pH=7.0)	Liang 2023[2]
pKa	-	8.99 (base)	Yamano 2001[3]
BRP	-	0.64	Liang 2023
Fup	-	0.30	Drugbank
P_{eff}	cm/s	0.58×10^{-4}	Liang 2023
CL_R	L/h	7.9	Moj 2017[4]
FPE (intestinal)	%	25	Liang 2023

Enzyme Table				
CYP3A4	K_m	μmol/L	41	Liang 2023
	V_{max}	pmol/min/mg	260	
Interaction				
Ki_{CYP3A4}		μM	10	Wei 2023[5]
$fu_{mic,CYP3A4}$		-	0.87	
k_{inact}		1/min	0.0355	Moj 2017
$Ki_{P\text{-}gp}$		μM	4	Wei 2023

Table S2. Physicochemical and absorption, distribution, metabolism, and excretion (ADME) information on dabigatran etexilate and dabigatran.

Parameters	Unit	Value	Reference
Dabigatran	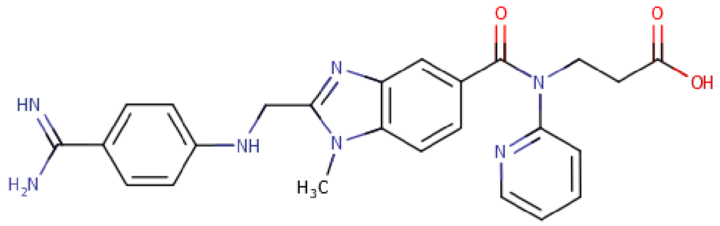		
Mw	g/mol	471.51	Drugbank
Solubility	mg/mL	0.017	2010[6]
LogP	-	2.2	Drugbank
Solubility	mg/mL	0.017	2010
pK_a	-	4.1 (base)<br>4.4 (base)<br>12.4 (base)	Moj 2019[7]
RBP	-	0.67	Moj 2019
Fup	-	0.65	Blech 2008[8]
P_{app}	cm/s	0.08×10^{-5}	PKsim
CL_R	L/h	7.1	Zhao 2014[9]
Enzyme Table			
UGT2B15 K_m	μmol/L	512	Stangier 2010[10]

	V_{max}	pmol/min/mg	31.8	Achour 2014[11]
	abun-dance	pmol/mg	62.1	
Dabigatran etexilate	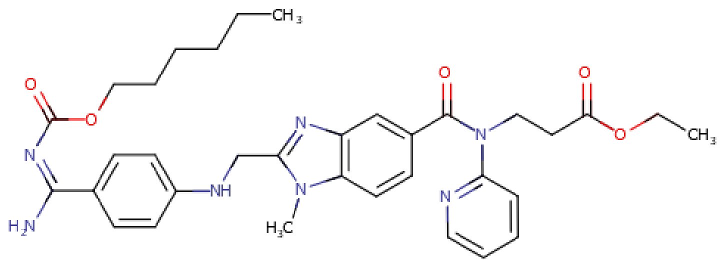			
Mw		g/mol	627	Drugbank
LogP		-	5.17	Drugbank
pK_a		-	4.0 (acid)<br>6.7 (base)	Zhao 2014
Solubility		mg/mL	1.8	Zhao 2014
BRP		-	3.08	Pksim
Fup		-	0.07	Moj 2019
P_{app}		cm/s	1.4×10^{-4}	Moj 2019
Enzyme Table				
CES 1	K_m	μmol/L	24.9	Moj 2019
	V_{max}	pmol/min/mg	676.0	
	abun-dance	pmol/mg	1664.4	
CES 2	K_m	μmol/L	5.5	
	V_{max}	pmol/min/mg	71.1	
	abun-dance	pmol/mg	174.1	
Transporter Table				
P-gp Gut	K_m	μmol/L	38.9	Doki 2019[12]
	V_{max}	pmol/min/million	146	

Table S3. Physicochemical and absorption, distribution, metabolism, and excretion (ADME) information on Rivaroxaban.

Parameters		Unit	Value	Reference
Structure			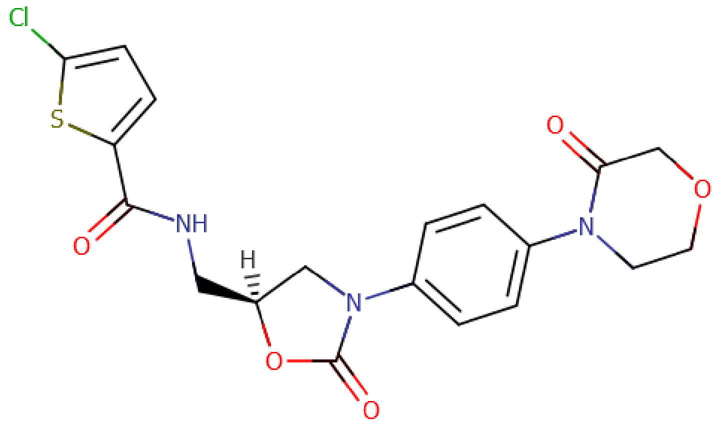	
Mw		g/mol	435.88	Cheong 2019[13]
LogP		-	1.5	Cheong 2019
pKa		-	-1.6 (base)<br>13.6 (acid)	Drugbank
Solubility		mg/mL	FaSSGf=0.0110 (pH 1.2)<br>FaSSIF=0.0168 (pH 5.0)<br>FeSSIF=0.0099 (pH 6.5)	Kushwah 2021[14]
BRP		-	0.71	Cheong 2019
Fup		-	0.65	Cheong 2019
Vss		L	50	Drugbank
P_{eff}		cm/s	3.020492×10^{-4}	Terrier 2023[15]
P_{app}		cm/s	4.74×10^{-6}	Kushwah 2021
CL_H		L/h	5.51	Cheong 2019
Enzyme Table				
CYP3A4	K_m	μmol/L	10	
	In vivo $CL_{int,u}$	L/h	76.363	Mueck 2013[16]
CYP2J2	K_m	μmol/L	10	
	In vivo $CL_{int,u}$	L/h	63.65	Mueck 2013
	Enzyme content	pmol/mg	1.2±2.1	Yamazaki 2006[17]

M_r	g/mol	5.77×10^4	King 2002[18]
Transporter Table			
P-gp K_m	μmol/L	9.416	Cheong 2019
P-gp V_{max}	pmol/min /million	80.921	Cheong 2019

Table S4. Physicochemical and absorption, distribution, metabolism, and excretion (ADME) information on Apixaban.

Parameters		Unit	Value	Reference
Structure		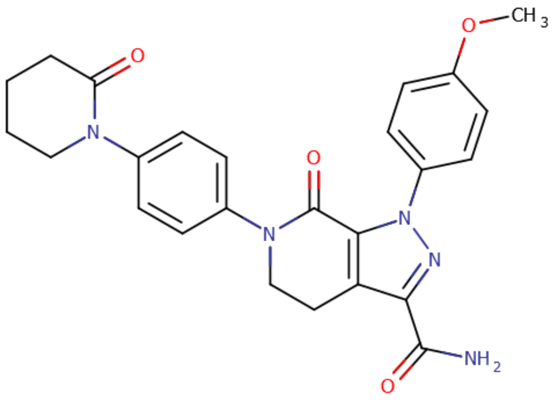		
Mw		g/mol	459.5	Drugbank
LogP		-	1.6	Drugbank
Solubility		mg/mL	0.11	Drugbank
BRP		-	0.9	ICRP.[19]
Fup		-	0.14	Drugbank
Vss		L	8	Terrier 2023[15]
P_{app}		cm/s	0.69×10^{-5}	Xu 2021[20]
$CL_{H/lint}$		L/h	2.8	Xu 2021
Enzyme Table				
CYP3A4	K_m	μmol/L	20	
	V_{max}	pmol/min/mg	40	
Transporter Table				Li 2023[21]
P-gp PBPK	K_m	μmol/L	99.1	
	V_{max}	mg/s	0.00135	

P-gp Gut	K_m	μmol/L	99.1
	V_{max}	mg/s	0.0099

Table S5. Physicochemical and absorption, distribution, metabolism, and excretion (ADME) information on Edoxaban.

Parameters	Unit	Value	Reference
Structure	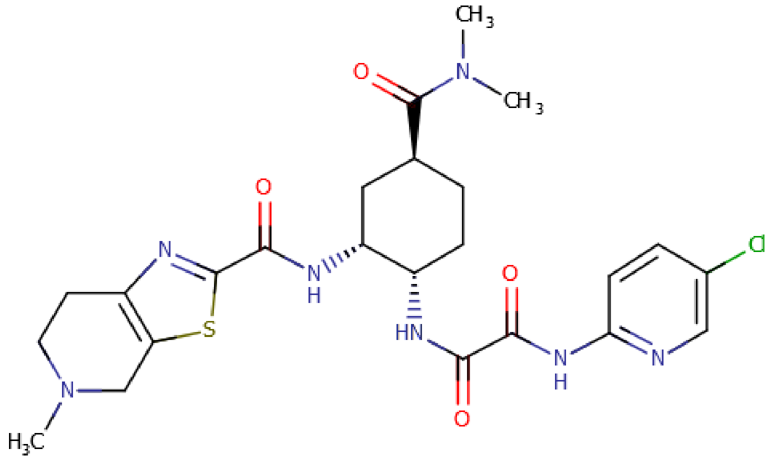		
Mw	g/mol	548	Drugbank
LogP	-	1.72 (pH=8)	Matsushima 2013[22]
Solubility	mg/mL	0.14 (pH=7)	Matsushima 2013
pK_a		10.34 (acid)<br>6.33 (base)	Drugbank
BRP	-	0.96	Matsushima 2013
Fup	-	0.457	Xu 2023[23]
Vss	L	81	Xu 2023
P_{eff}	cm/s	2.06×10^{-4}	Matsushima 2013
Biliary CL fraction	% of $CL_{H, int}$	50	Matsushima 2013
CL_H	L/h	10.76	Matsushima 2013
CL_R	L/h	10.7	Matsushima 2013
Enzyme Table			
CYP3A4 K_m	mg/L	100	Matsushima 2013
CYP3A4 V_{max}	mg/s/mg	0.0006	Matsushima 2013

Transporter Table				
P-gp Gut	K_m	mg/L/	5.92	Matsushima 2013
	V_{max}	mg/s/mg	0.0132	

Table S6. Comparisons between PBPK model predictions and reported clinical data collated from the literature.

Predicted value		DAB	Rivaroxaban	Apixaban	Edoxaban
		Mean	Mean	Mean	Mean
DDI-Cmax (ng/mL)	Observed	294	194	339	258
	Predicted	359	152	355	293
	Ratio	1.22	0.78	1.05	1.14
Baeseline-Cmax (ng/mL)	Observed	174	139	261	203
	Predicted	249	108	240	198
	Ratio	1.39	0.78	0.92	0.98
DDI-AUC (ng·h/mL)	Observed	1820.4	1649	4036	1783
	Predicted	3614.6	1233.7	3972.0	2022.8
	Ratio	1.98	0.75	0.98	1.13
Baseline-AUC (ng·h/mL)	Observed	1220.5	964	2531	1166
	Predicted	2532.9	594.2	2129.3	1047.7
	Ratio	2.00	0.62	0.84	0.90

References

1. Lappin G, Shishikura Y, Jochemsen R, et al. Comparative pharmacokinetics between a microdose and therapeutic dose for clarithromycin, sumatriptan, propafenone, paracetamol (acetaminophen), and phenobarbital in human volunteers. *Eur J Pharm Sci*. 2011;43(3):141-150. doi：10.1016/j.ejps.2011.04.009.
2. Liang N, Zhou S, Li T, et al. Physiologically based pharmacokinetic modeling to assess the drug-drug interactions of anaprazole with clarithromycin and amoxicillin in patients undergoing eradication therapy of H. pylori infection. *Eur J Pharm Sci*. 2023;189:106534. doi：10.1016/j.ejps.2023.106534.
3. Yamano K, Yamamoto K, Katashima M, et al. Prediction of midazolam-CYP3A inhibitors interaction in the human liver from in vivo/in vitro absorption, distribution, and metabolism data. *Drug Metab Dispos*. 2001;29(4 Pt 1):443-452. doi：10.3390/ph16030360.
4. Moj D, Hanke N, Britz H, et al. Clarithromycin, Midazolam, and Digoxin: Application of PBPK Modeling to Gain New Insights into Drug-Drug Interactions and Co-medication Regimens. *Aaps J*. 2017;19(1):298-312. doi：10.1208/s12248-016-0009-9.
5. Wei Z, Jeong HC, Kim MG, et al. Prediction of the Drug-Drug Interaction Potential between Tegoprazan and Amoxicillin/Clarithromycin Using the Physiologically Based Pharmacokinetic and Pharmacodynamic Model. *Pharmaceuticals (Basel)*. 2023;16(3). doi：10.3390/ph16030360.
6. Boehringer Ingelheim Pharmaceuticals Inc. Dabigatran Etexilate __Capsules (75 mg, 110 mg, and 150 mg). 2010.

7. Moj D, Maas H, Schaeftlein A, et al. A Comprehensive Whole-Body Physiologically Based Pharmacokinetic Model of Dabigatran Etexilate, Dabigatran and Dabigatran Glucuronide in Healthy Adults and Renally Impaired Patients. *Clinical Pharmacokinetics*. 2019;58(12):1577-1593. doi：10.1007/s40262-019-00776-y.
8. Blech S, Ebner T, Ludwig-Schwellinger E, et al. The metabolism and disposition of the oral direct thrombin inhibitor, dabigatran, in humans. *Drug Metab Dispos*. 2008;36(2):386-399. doi：10.1124/dmd.107.019083.
9. Zhao Y, Hu ZY. Physiologically based pharmacokinetic modelling and in vivo [I]/K(i) accurately predict P-glycoprotein-mediated drug-drug interactions with dabigatran etexilate. *Br J Pharmacol*. 2014;171(4):1043-1053. doi：10.1111/bph.12533.
10. Stangier J, Rathgen K, Stahle H, et al. Influence of renal impairment on the pharmacokinetics and pharmacodynamics of oral dabigatran etexilate: an open-label, parallel-group, single-centre study. *Clin Pharmacokinet*. 2010;49(4):259-268. doi：10.2165/11318170-000000000-00000.
11. Achour B, Russell MR, Barber J, et al. Simultaneous quantification of the abundance of several cytochrome P450 and uridine 5'-diphospho-glucuronosyltransferase enzymes in human liver microsomes using multiplexed targeted proteomics. *Drug Metab Dispos*. 2014;42(4):500-510. doi：10.1124/dmd.113.055632.
12. Doki K, Neuhoff S, Rostami-Hodjegan A, et al. Assessing Potential Drug-Drug Interactions Between Dabigatran Etexilate and a P-Glycoprotein Inhibitor in Renal Impairment Populations Using Physiologically Based Pharmacokinetic Modeling. *Cpt-Pharmacomet Syst*. 2019;8(2):118-126. doi：10.1002/psp4.12382.
13. Cheong EJY, Teo DWX, Chua DXY, et al. Systematic Development and Verification of a Physiologically Based Pharmacokinetic Model of Rivaroxaban. *Drug Metab Dispos*. 2019;47(11):1291-+. doi：10.1124/dmd.119.086918.
14. Kushwah V, Arora S, Tamas Katona M, et al. On Absorption Modeling and Food Effect Prediction of Rivaroxaban, a BCS II Drug Orally Administered as an Immediate-Release Tablet. *Pharmaceutics*. 2021;13(2). doi：10.3390/pharmaceutics13020283.
15. Terrier J, Gaspar F, Gosselin P, et al. Apixaban and rivaroxaban's physiologically-based pharmacokinetic model validation in hospitalized patients: A first step for larger use of a priori modeling approach at bed side. *Cpt-Pharmacomet Syst*. 2023;12(12):1872-1883. doi：10.1002/psp4.13036.
16. Mueck W, Kubitza D, Becka M. Co-administration of rivaroxaban with drugs that share its elimination pathways: pharmacokinetic effects in healthy subjects. *Brit J Clin Pharmaco*. 2013;76(3):455-466. doi：10.1111/bcp.12075.
17. Yamazaki H, Okayama A, Imai N, et al. Inter-individual variation of cytochrome P4502J2 expression and catalytic activities in liver microsomes from Japanese and Caucasian populations. *Xenobiotica*. 2006;36(12):1201-1209. doi：10.1080/00498250600944318.
18. King LM, Ma JX, Srettabunjong S, et al. Cloning of CYP2J2 gene and identification of functional polymorphisms. *Mol Pharmacol*. 2002;61(4):840-852. doi：10.1124/mol.61.4.840.
19. ICRP. Basic anatomical and physiological data for use in radiological protection: reference values. A report of age- and gender-related differences in the anatomical and physiological characteristics of reference individuals. ICRP Publication 89. *Ann ICRP*. 2002;32(3-4):5-265.
20. Xu R, Tang H, Chen L, et al. Developing a physiologically based pharmacokinetic model of apixaban to predict scenarios of drug-drug interactions, renal impairment and paediatric populations. *Br J Clin Pharmacol*. 2021;87(8):3244-3254. doi：10.1111/bcp.14743.
21. LI Yamei XP, ZHANG Yu ,CHEN Zhixing ,SHI Aixin. Quantitative Analysis of the Effect of P-Glycoprotein on Apixaban Disposal Based on Physiological Pharmacokinetic Model. 2023;58(15):1384-1390. doi：10. 11669 /cpj. 2023. 15. 006.
22. Matsushima N, Lee F, Sato T, et al. Bioavailability and Safety of the Factor Xa Inhibitor Edoxaban and the Effects of Quinidine in Healthy Subjects. *Clin Pharmacol Drug Dev*. 2013;2(4):358-366. doi：10.1002/cpdd.53.
23. Xu R, Liu W, Ge W, et al. Physiologically-based pharmacokinetic pharmacodynamic parent-metabolite model of edoxaban to predict drug-drug-disease interactions: M4 contribution. *CPT Pharmacometrics Syst Pharmacol*. 2023;12(8):1093-1106. doi：10.1002/psp4.12977.
